# Using environmental DNA methods to improve detectability in an endangered sturgeon (*Acipenser sinensis*) monitoring program

**DOI:** 10.1186/s12862-021-01948-w

**Published:** 2021-12-01

**Authors:** Dan Yu, Zhongyuan Shen, Tao Chang, Sha Li, Huanzhang Liu

**Affiliations:** 1 The Key Laboratory of Aquatic Biodiversity and Conservation of Chinese Academy of Sciences, Institute of Hydrobiology, Chinese Academy of Sciences, Wuhan, 430072 Hubei China; 2 University of Chinese Academy of Sciences, Beijing, 100049 China; 3 Hubei Key Laboratory of Three Gorges Project for Conservation of Fishes, Chinese Sturgeon Research Institute, China Three Gorges Corporation, Yichang, 443100 Hubei China

**Keywords:** Environmental DNA, Sturgeon, Reproductive stock, Droplet digital PCR

## Abstract

**Background:**

To determine the presence and abundance of an aquatic species in large waterbodies, especially when populations are at low densities, is highly challenging for conservation biologists. Environmental DNA (eDNA) has the potential to offer a noninvasive and cost-effective method to complement traditional population monitoring, however, eDNA has not been extensively applied to study large migratory species. Chinese sturgeon (*Acipenser sinensis*), is the largest anadromous migratory fish in the Yangtze River, China, and in recent years its population has dramatically declined and spawning has failed, bringing this species to the brink of extinction. In this study, we aim to test the detectability of eDNA methods to determine the presence and relative abundance of reproductive stock of the species and whether eDNA can be used as a tool to reflect behavioral patterns. Chinese sturgeon eDNA was collected from four sites along the spawning ground across an eight month period, to investigate the temporal and spatial distribution using droplet digital PCR (ddPCR).

**Results:**

We designed a pair of specific primers for Chinese sturgeon and demonstrated the high sensitivity of ddPCR to detect and quantify the Chinese sturgeon eDNA concentration with the limit of detection 0.17 copies/μl, with Chinese sturgeon eDNA been intermittently detected at all sampling sites. There was a consistent temporal pattern among four of the sampling sites that could reflect the movement characteristics of the Chinese sturgeon in the spawning ground, but without a spatial pattern. The eDNA concentration declined by approximately 2–3 × between December 2018 and December 2019.

**Conclusions:**

The results prove the efficacy of eDNA for monitoring reproductive stock of the Chinese sturgeon and the e decreased eDNA concentration reflect that Chinese sturgeon may survive with an extremely small number of reproductive stock in the Yangtze River. Accordingly, we suggest future conservation measures should focus on both habitat restoration and matured fish restocking to ensure successful spawning. Overall, this study provides encouraging support for the application of eDNA methods to monitor endangered aquatic species.

**Supplementary Information:**

The online version contains supplementary material available at 10.1186/s12862-021-01948-w.

## Background

Determining the presence and estimating the abundance of organisms is critical to the study and conservation of endangered species. However, estimating the organism abundance with accuracy and precision is often biased, invasive or costly, particularly in aquatic systems where organisms are hidden underwater. The emergence of environmental DNA (eDNA) has greatly improved detection of aquatic organisms, particularly for rare or endangered species [1–5]. The DNA that is shed or excreted from individuals during normal activity can be collected, extracted and amplified via the Polymerase Chain Reaction (PCR) to reveal the habitat’s species composition [6, 7]. At present, eDNA metabarcoding has been used widely to detect various taxa, including invertebrates [8, 9], fish [10–12], amphibians [13, 14] and mammals [15, 16], as well as in diverse ecosystems such as lakes [17, 18], rivers [19, 20] and oceans [21, 22]. Recently, several studies have demonstrated the robust efficiency of eDNA in detecting a rare target species from other closely related species and endangered species, e.g. Wilcox et al. [23] successfully detected the rare bull trout (*Salvelinus confluentus*) from brook trout (*Salvelinus fontinalis*) and Qu et al. [24] found eDNA less expensive in monitoring the critically endangered mammal, Yangtze finless porpoise (*Neophocaena asiaeorientalis asiaeorientalis*) than traditional methods. However, the majority of eDNA studies have focused on the occurrence of target species, not quantifying abundance or biomass [25, 26]. Consequently, development of eDNA to estimate species’ abundance, will greatly expand the applicability of this technology for future conservation and management [27, 28].

Recently, some studies started to use quantitative PCR (qPCR) to demonstrate significant correlation between DNA estimates and abundance or biomass [5, 29–33], indicating the potential to infer relative population size from such methods. However, the ability to accurately and precisely estimate organism abundance from quantified eDNA remains largely uncertain outside of mesocosms [34]. Most quantitative eDNA studies found that eDNA concentration explains on average ~ 80% of the variability in organism abundance in controlled mesocosms, but only ~ 50% across natural systems [34]. Although the explanatory power of eDNA in natural systems needs to continue to improve, quantitative eDNA is still a useful tool for monitoring programs.

A new PCR method for quantifying low levels of DNA has been developed, droplet digital PCR (ddPCR), fractionating a PCR reaction into more than 20,000 droplets using an oil emulsion [35], some of which ideally contain one or a few copies of the target DNA. Amplification of the target DNA is quantified by incorporating a fluorescent dye directly into the amplicon reaction. The target-positive and target-negative droplets are individually counted by passing them in a single stream through a fluorescence detector, with their ratio used to estimate the number of copies of the target DNA in the sample. Thus, ddPCR allows for direct quantification without making standard curves that are necessary for qPCR [36]. Compared to qPCR, ddPCR had higher rates of positive detections in pond samples of Grass Carp (*Ctenopharyngodon idella*) due to increased sensitivity and dilution of inhibitors at low concentrations [37]. Recently, studies have suggested that ddPCR is better suited to measure eDNA presence-absence or eDNA concentration in water and provides more accurate results for the abundance of the target species than qPCR in fish [35, 37, 38]. Wood et al. (2019) [39] compared the sensitivity of qPCR and ddPCR to detect a specific-species *Sabella spallanzanii*, and the results found ddPCR copy numbers were on average 125-fold higher than those measured using qPCR and the detection probabilities of ddPCR (1.0) were higher than qPCR (0.93). However, few quantitative ddPCR studies have been applied to estimate the abundance of endangered large migratory species (see Baker et al. [40]).

Chinese sturgeon (*Acipenser sinensis* Gray, 1835) is the largest anadromous migratory fish in the Yangtze River and was categorized as a critically endangered species by the IUCN in 2010. In summer, breeding Chinese sturgeon adults start to migrate from the near shore areas of the Yellow and East China Sea’s to the Yangtze River and stay in the Yangtze River for one year to reach sexual maturity and spawn during the next autumn [41]. Since 1981, the construction of the Gezhou Dam has blocked their migration route and the only available spawning ground was limited to a narrow reach of three kilometers downstream of the dam [42]. Moreover, the impoundments of the Three Gorges Reservoir in 2003 have further deteriorated the spawning habitat and increasingly threatening the survival of Chinese sturgeon [43]. Under these circumstances, the population size of Chinese sturgeon has been continuously declining. In recent years (2013, 2015, 2017–2019), there has been no evidence that the Chinese sturgeon had spawned below the Gezhou Dam, suggesting this species is already on the brink of extinction in the wild [44]. In order to assess this species’ status and take effective managements and conservation measures, it is pressing to make consecutively accurate population monitorings.

Some researchers have demonstrated the ability of eDNA to detect the presence of sturgeons [45–48]. Determining sturgeon presence is important, but alone this might provide little information on population status. However, analysis of eDNA concentrations through ddPCR may allow researchers to track and reflect on the movement route of the adults or their reproductive activities. In this study, we test the detectability of eDNA using ddPCR to detect and quantify the Chinese sturgeon along the spawning ground in the Yangtze River, China. Specifically, we had two objectives: 1) Determine the Chinese sturgeon eDNA presence and concentration along the spawning ground; 2) Collect water samples over eight months from four sites to examine whether eDNA exhibit special temporal and spatial patterns, and whether eDNA can be used to reflect behavioral patterns of Chinese sturgeon. Our analyses may provide important data on which future conservation of endangered species might be based, as well as a general method for the monitoring of endangered aquatic species.

## Materials and methods

### eDNA sampling

**Fig. 1:**
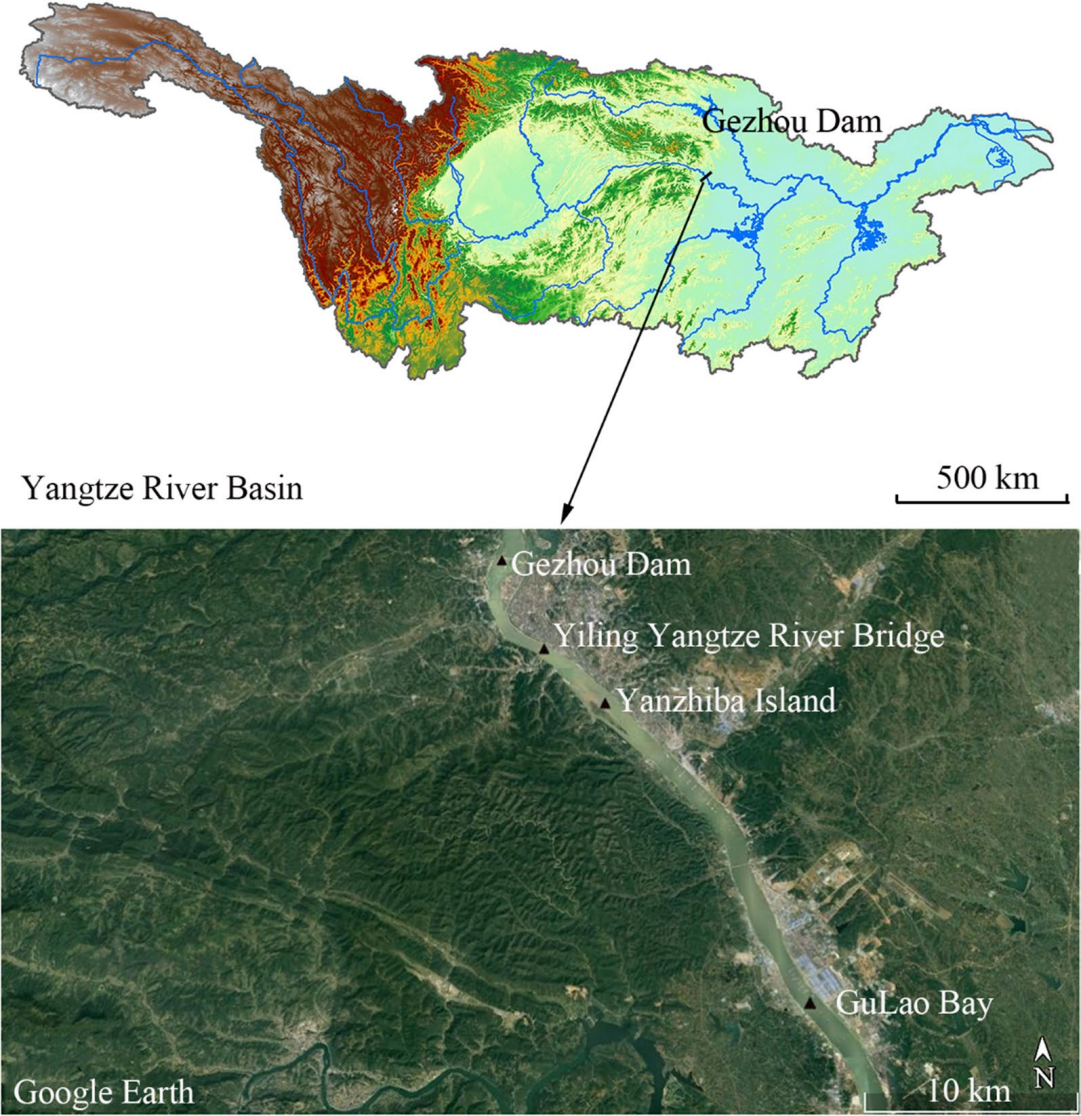
Sampling collection sites for eDNA in the Yangtze River Basin. ArcGIS 10.2 and Google Earth was used to produce a distribution map

We identified and set four sites to collect eDNA samples according to the historical records of spawning sites since the establishment of Gezhou Dam in 1981 (Fig. 1). The width of four sampling sites are 850 m (Gezhou Dam), 580 m (Yangtze Bridge), 1100 m (Yanzhi Dam) and 750 m (Gulaobei Bay). The eDNA samples were collected once a month during the eight months of December 2018, June and August to December 2019, and January 2020. Since July is the annual flood season in the middle and lower reaches of the Yangtze River, the samples in July were not collected. At each site, three replicate 2L water samples were collected from 6 to 10 m below the water surface, dozens of meters away from the shoreside with a polyvinyl chloride water sampler and immediately transferred into the sterilized bottles surrounded with ice. Water samples were stored on ice before filtration while avoiding exposure to light to prevent eDNA degradation. A total of 96 water samples (3 replicates over 4 sites during 8 months) were collected. We assessed potential cross-contamination by filling one bottle with deionized water before each sampling, opening and closing in the field as blanks (32 collections consisting of, 1 blank × 4 sites × 8 months), and treated all the blanks with the same procedures as other samples. All working surfaces and equipment (e.g., bottles and samplers) were decontaminated using 10% bleach before and after each sample collection including in the same sampling location during the same sampling event.

### Filtration of eDNA samples

Each eDNA water sample was filtered on a vacuum filtration pump (Tianjin Jinteng Experimental Equipment Co., Lot) fitted with a 47 mm diameter cellulose nitrate filter membrane with 0.45 μm pores (Whatman, Maidstone, UK). To exclude additional contamination during filtration and extraction, we filtered deionized water as negative controls that were subsequently extracted and amplified. Meanwhile, to reduce DNA degradation, the filtration processing was finished within four hours after collection. Only one membrane was filtered for each water sample and immediately placed into a sterilized centrifuge tube with sterile forceps and stored at -80 °C until used for DNA extraction.

### eDNA extraction and primer design

The eDNA was extracted from filtered membranes following the standard protocol of the DNeasy® PowerWater® DNA Isolation Kit (Qiagen). Before DNA extraction, we ripped each filtered membrane into a few pieces within a sterile tube. Extraction negative controls (consisting of the same extraction materials with no filter, replaced with nuclease-free water) were run for each extraction batch (n = 32) to monitor for any contamination. To optimize the detection and identification, we designed a pair of specific diagnostic primers for Chinese sturgeon from the D-loop region on the mitochondrial DNA genome. Primers were designed using Primer-BLAST [49] and checked for potential cross-amplifications in syntopic taxa (Additional file 1: Appendix S1). The primers amplifie a fragment of 132 bp in length and sequences of primers are shown as below:

- Forward primer 5′ GGCAATTTTAATCTGGGTTTCCA 3′;
- Reverse primer 5′ TGGATGTTAGATATATGTCCTTG 3′.

### ddPCR and limit of detection

The Chinese sturgeon eDNA was detected and quantified by a Bio-Rad QX200™ AutoDG™ Droplet Digital™ PCR System at the Analysis and Testing Center in Institute of Hydrobiology, Chinese Academy of Sciences. We performed the ddPCR amplification in 20 μl reaction volume containing 2 μl water sample genomic DNA, 2 μl of each primer (1 μM), 4 μl sterile deionized water and 10 μl 1 × supermix for EvaGreen (Bio-Rad), which was mixed with Bio-Rad droplet generator oil and partitioned into approximately 20,000 droplets. Samples were run with the following thermocycling profile: initial denaturation at 95 °C for 10 min followed by 30 cycles of denaturation at 94 °C for 30 s, annealing at 55 °C for 1 min, and holding at 4 °C. After PCR amplification, the PCR products were transferred to the Bio-Rad QX-100 droplet reader. Each sample mixed with droplets was checked for fluorescence to count the number of droplets that yielded positive or negative results. The result was calculated by the Bio-Rad’s QuantaSoft software. Quantification of target DNA, in copies/μl of reaction, is based on an assumption of a Poisson distribution of the target DNA among the 20,000 droplets [50].

We firstly conducted a serial dilution of positive controls to assess the relative sensitivity of ddPCR. The initial sample was a standard DNA extracted from the Chinese sturgeon tail fin. The first dilution was 1/2 of the initial sample, and the second one was 1/4 and so on, ultimately producing 13 dilutions (1/2 to 1/8192). These dilutions were used as templates to detect the reliability and define the limit of detection within each reaction.

Secondly, the ddPCR was run in all eDNA samples, negative and positive controls (standard samples). The Chinese sturgeon was considered present at the site if two out of three samples could amplify more positive production than the detection limit. Samples that were lower than the detection limit, but higher than zero, were considered ambiguous to determine. However, negative or ambiguous samples would be tested again.

After finishing the ddPCR reaction, we randomly selected and re-amplified one positive ddPCR product at each site (four samples in total) and two standard Chinese sturgeon DNA samples, and their productions were then sequenced using sanger sequencing to ensure the amplifications were specific from the Chinese sturgeon. Finally, we constructed phylogenetic relationships combining the above six sequences with 18 sequences of sturgeons and paddlefishes using unweighted pair-group method with arithmetic mean (UPGMA) in MEGA X [51].

### Temporal and spatial distribution for eDNA

According to historical records of reproductive activities [44], we divided the sampling period into three seasons: pre-breeding (the months from June to October represent the time before breeding), breeding (November and December are the months when the breeding activities take place) and post-breeding (January represents the time after breeding). To examine whether eDNA distributions and concentrations were influenced by either season or sampling site, we used the mixed effects model with season, sampling site and the interaction of these variables as fixed effects. The sampling time and site were included as random effects and modeled as random slopes based on the fixed effects. Mixed effects model was conducted using the ‘lme4’ package [52] in R v2.15.1 [53] and models were ranked based on AIC value.

## Results

### Primer specificity and limit of detection for ddPCR

We performed sequence match and phylogenetic relationship analysis to confirm the primers’ specificity. The sequences of two primers provided a perfect sequence match with the D-loop sequence of the Chinese sturgeon (GenBank accession number: AY096295; see Additional file 1: Appendix S1). The phylogenetic relationship also showed that sequences of four eDNA positive ddPCR product could be distinguished clearly from other species, even compared with the closest related species *Acipenser dabryanus*, a potential sympatric species in Yangtze River (Additional file 1: Appendix S2). Therefore, the primers were Chinese sturgeon-specific.

**Fig. 2:**
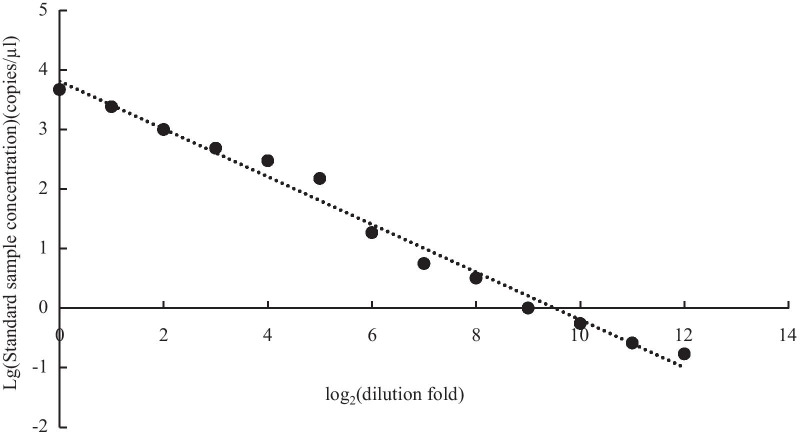
Analysis of correlation between the logarithmic transformations of ddPCR concentration and dilution fold

To assess the sensitivity of ddPCR, we conducted a serial dilution of positive controls using the Chinese sturgeon standard DNA incorporated with the designed primers. The ddPCR concentration of the initial sample reached 4,680 copies/μl and the lowest limit of detection was 0.17 copies/μl at a dilution of 1/4096. There would be no detectable DNA at the dilution of 1/8192. A significant linear relationship (*R*^2^ = 0.986, *p* < 0.01; Fig. 2) was tested between both the logarithmic transformations of ddPCR concentration and dilution fold. This relationship indicated that ddPCR was sensitive to quantify the Chinese sturgeon eDNA from water samples and those samples with ddPCR concentration > 0.17 copies/μl could be detected accurately.

### Presence and absence of the Chinese sturgeon eDNA

**Fig. 3:**
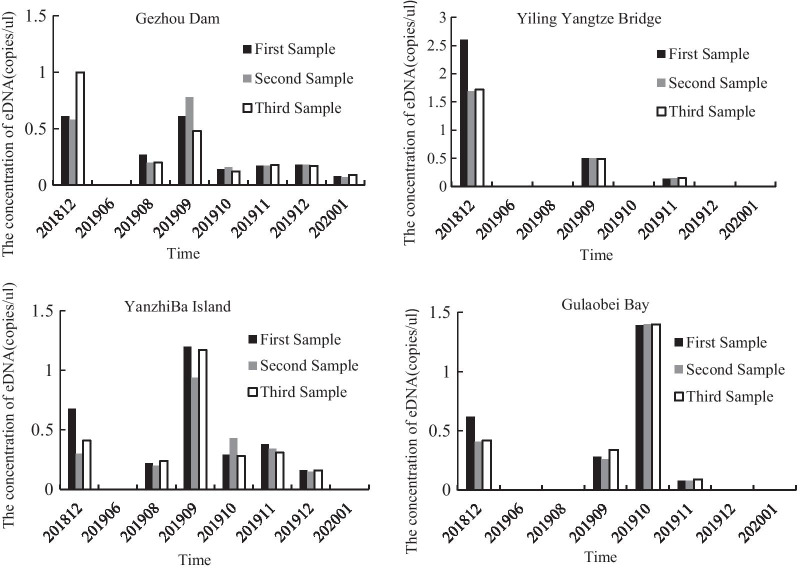
Estimates of Chinese sturgeon eDNA quantity across eight months at four sampling sites

We evaluated presence and absence of the Chinese sturgeon in each water sample using ddPCR. All sampling blanks, extraction and no-template ddPCR controls were negative for the Chinese sturgeon DNA. The Chinese sturgeon eDNA was present in 48 water samples (50.00%), ambiguous in 15 water samples (15.63%), and absent in 33 water samples (34.37%). In terms of the whole sampling period, the Chinese sturgeon eDNA has been intermittently detected at all sampling sites. The majority of absence records were found in June 2019 and January 2020, and other months changing patterns would be discussed below (Table 1; Fig. 3; Additional file 1: Appendix S3). These results showed that the Chinese sturgeon had been present along the spawning ground area in 2018 and 2019.

**Table 1:** The ddPCR concentrations of Chinese sturgeon eDNA from four sites across eight months

Time	Gezhou Dam (copies/µl)	Yiling Yangtze River Bridge (copies/µl)	YanzhiBa Island (copies/µl)	Gulaobei Bay (copies/µl)
2018/12	0.73	2.00	0.46	0.48
2019/06	0.00	0.00	0.00	0.00
2019/08	0.22	0.00	0.22	0.00
2019/09	0.62	0.50	1.10	0.29
2019/10	0.14	0.00	0.33	1.40
2019/11	0.17	0.15	0.34	0.08
2019/12	0.18	0.00	0.16	0.00
2020/01	0.08	0.00	0.00	0.00

Each time-site value represents the average result of three replicates

### Temporal and spatial distribution of the Chinese sturgeon eDNA

To test whether eDNA distributions and concentrations were influenced by either season or sampling site, we used mixed effects model with season, site and the interaction of these variables as fixed effects. The best supported model with an AIC value of 50.07 included season as a fixed effect and a random slope effect based on time and season (Table 2). Sampling sites showed the least effect on eDNA distribution with the highest AIC value of 80.58. Thus, the major difference based on season was clearly during the sampling period (Table 2; Fig. 3), as each site had elevated levels in September or October that returned to low levels in November and December (Table 1; Fig. 3; Additional file 1: Appendix S3). In pre-breeding season, the target eDNA was absent in June, indicating that Chinese sturgeon were not present in these areas before breeding season. Then the eDNA concentration was increased from August to September or October, with the peak occurring at Gezhou Dam and Yiling Yangtze Bridge in September while at YanzhiBa Island and Gulaobei Bay in October. Notably, the eDNA concentrations decreased rapidly at all sampling sites in breeding season (November and December), indicating some individuals had begun to leave the spawning ground, even before the breeding season had not ended, probably due to no suitable spawning conditions. In post-breeding season, eDNA almost disappeared at all sampling sites, which reflects that Chinese sturgeon adults probably migrate to other reaches due to no suitable spawning ground or because of the eDNA degradation. Taken together, these results strongly suggest the variation of eDNA concentration is a true reflection of movement characteristics of the Chinese sturgeon and shows a clear temporal pattern.

**Table 2:** AIC model selection results for the three Chinese sturgeon eDNA models

Model	AIC value
Season + (season|time)	50.0687
Season + (season|time) + (season|site)	56.0689
Season + site + (season|site) + (site|site)	80.5791

Season is divided into three categories (pre-breeding, breeding, post-breeding), time and site representing the sampling time and locality

As the mixed effects model tested, sampling sites contributed the least effect on eDNA distribution, and indeed no obvious spatial pattern could be found among sampling sites (Fig. 3). In 2019, the most abundant eDNA was detected from Gulaobei Bay in October (1.4 copies/µl) while there was no eDNA at Yiling Yangtze Bridge (Fig. 3; Additional file 1: Appendix S3). In addition, the Chinese sturgeon eDNA from Gezhou Dam and YanzhiBa Island shared a similar eDNA concentration between the two sites in every month.

## Discussion

Our results present a successful ddPCR-based method to detect the Chinese sturgeon eDNA along the spawning ground in the Yangtze River, China. The demonstrated high level of sensitivity conclude that eDNA surveys can complement traditional surveys in monitoring programs. In addition, there was a consistent temporal pattern among four sampling sites that showed eDNA concentration increased in September or October 2019, but no obvious spatial pattern. Finally, we interpret the temporal and spatial patterns in eDNA concentration in association with the Chinese sturgeon’s movement characteristics.

### High level of sensitivity of ddPCR

We demonstrated the potential of ddPCR to detect and quantify the Chinese sturgeon eDNA concentration. Each positive eDNA sample was determined from three replicates (Fig. 3; Additional file 1: Appendix S3), demonstrating very accordant amplification within a sampling site. This is consistent with the findings of Xu et al. [47] whose study successfully detected presence of Chinese sturgeon using eDNA from Yichang site during the spawning season. However, their research only used conventional PCR to test for presence and absence for this species, whereas we tested for sensitive PCR (ddPCR) to get the quantitative information of eDNA which may related with the abundance of spawning individuals. For the Chinese sturgeon eDNA, the threshold of > 0.17 copies/μl was well supported by the detection limits analysis, indicating the high detectability. This threshold is consistent with the limit of detection in Grass Carp (0.13 copies/μl [37],) and whales (0.12 copies/μl [40],), suggesting our ddPCR detection is of high level of sensitivity even at low concentrations. These results support the conclusion that the efficiency and effectiveness of ddPCR for detection of Chinese sturgeon eDNA.

### Temporal and spatial distribution of the Chinese sturgeon eDNA

An obvious temporal pattern has been found that a marked eDNA concentration increase was presented in September or October 2019, compared to other months before and after. Although the magnitude of increase differed among sites, each site showed increased eDNA during this time. Whilst anticipated, we speculate this increase in DNA is related to the movement characteristics during spawning season, as the Chinese sturgeon adults need to find a suitable egg-laying sites for spawning [43]. However, their relationships should be further verified in the future when the abundance estimation based on traditional surveys are simultaneously available with the same sampling frequency. In turn, the surge in eDNA concentration suggests the presence of individuals in reproductive condition may be sufficient to cause this peak even without actual reproductive activities (no egg was found in 2018 and 2019 through investigating stomach and intestine of benthic carnivorous fishes, see Bulletin on the Ecological and Environmental Monitoring Results of the Three Gorges Project). Similar phenomenon has been reported in hellbender that eDNA presented at the highest level but without real reproductive behaviors [54]. Temporal variation of eDNA concentration has been found in some other studies [17, 55], demonstrating there might be an optimum sampling time for species of interest that will be used to ensure maximum detection. In this study, the consistent mounts in eDNA concentration indicate that September or October may be the optimal time to collect eDNA water samples when attempting to monitoring reproductive stock of Chinese sturgeon along the spawning ground.

According to our results, there was no consistent spatial pattern among four sampling sites. Although the most abundant eDNA was detected in Gulaobei Bay in October, the most downstream site, few general trends have been found in other months. This lack of a consistent spatial pattern in eDNA concentrations in multiple sites along the same river has also been observed in a migratory Chinook salmon (*Oncorhynchus tshawytscha*, [55]) and amphibians [54]. The migratory behaviors may be the main reason for the absence of consistent spatial pattern. However, in some resident or non-migratory species, more positive relationships were prone to detect between eDNA abundances and spatial distribution of population densities. For example, eDNA concentrations of common carp (*Cyprinus carpio*) were strongly correlated with its population density distribution in a lake [56]. Besides, the relationship between eDNA abundances and spatial distribution of a species may be complex and confounded by various biological and environmental factors, especially in a lotic system [57, 58]. First, the uncertainty of eDNA quantification was increased because eDNA estimates are influenced by individuals that occur upstream of the sampling site [59, 60]. For example, Kessler et al. [61] demonstrated that eDNA methods could be used to detect a benthic turtle species (*Macrochelys temminckii*) in a lotic system but that eDNA presence was dependent upon upstream biomass, and detection could be affected under UV exposure. Second, other environmental factors such as river depth, temperature and water flow dynamics may also influence the eDNA quantification. Song et al. [62] revealed that stream flow direction was highly influential on eDNA detection in the Chicago AreaWaterway System. Furthermore, there can be more variation on a day to day basis (e.g., due to high rainfall events) than across months or seasons. However, the relationship between water current and eDNA transport is not well known. Therefore, further research is needed to better understand the ecology of eDNA transport.

### Cost and effort comparison between eDNA detection and hydro-acoustic telemetry

Several studies have evaluated the methodological efficiency of eDNA versus traditional surveys in detecting rare and endangered species, and demonstrated that the detectability of eDNA is relatively higher than or comparable to that of traditional surveys [63]. For instance, when monitoring a critically endangered mammal in the Yangtze River, Yangtze finless porpoise, Qu et al. [24] found visual surveying on a monthly basis costed 1.41 × that of eDNA collections utilizing qPCR at the same temporal schedule. Similarly, assessing the distribution of Brook Trout (*Salvelinus fontinalis*) using eDNA analysis required lower sampling effort and 67% less cost than triple-pass electrofishing [64]. Here, we simply compared cost and effort between eDNA detection and traditional hydro-acoustic telemetry survey for the Chinese sturgeon monitoring work. Environmental DNA sampling mainly included boat rentals ($ 300/day), labor cost ($ 80/day) for three days and laboratory consumption such as filtering, extraction and screening the samples with ddPCR ($ 8/sample), however, the eDNA sampling only took place once a month. Therefore, the overall cost of analyzing our 12 eDNA samples and four control samples per month was about $ 1, 268. Hydro-acoustic telemetry survey was normally conducted seven days per month and the costs and efforts were focused on boat rentals ($ 300 /day) and a two-person-team labor ($ 160 /day), approximated to $ 3, 220 per month. It is definitely clear that the monetary costs and sampling efforts spent on eDNA are much less than hydro-acoustic telemetry survey. Therefore, our result is consistent with previous studies.

### The low abundance and conservation

The eDNA concentration at Gezhouba Dam (December 2019, 0.73 copise/µl) decreased by about 75% comparing with that in December 2018 (0.18 copies/µl). At YanzhiBa Island, the eDNA concentration similarly decreased by about 67% (December 2019, 0.46 copies/µl; December 2018, 0.16 copies/µl, Table 1). This approximate 2–3 × decline may suggest the Chinese sturgeon eDNA continue to decline in the Yangtze river year by year. These results are consistent with previous studies that the number of Chinese sturgeon has being rapidly decreasing since the construction of Gezhou Dam [44, 65] and now an extremely small number of reproductive stock exist in the spawning ground. Although it could be sensitively detected, the eDNA concentration around the spawning ground is approximate to zero, indicating that the reproductive stock of Chinese sturgeon is rather small and might be on the brink of extinction. Therefore, we suggested increasing the eDNA survey frequency in future monitoring work.

In recent years (2013, 2015 and 2017–2019), the Chinese sturgeon failed to spawn in the Yangtze River (Bulletin on the Ecological and Environmental Monitoring Results of the Three Gorges Project). Chang et al. (2021) [66] recently analysed hydrological regime requirements for spawning success of the Chinese sturgeon and discussed the causes of spawning failure. They found that the long duration (> 100 days) and large magnitude (> 1.8*10^6^ m^3^/s) of high flow pulse in the flood season and low accumulated water temperature in October (< 660 °C) and November (< 540 °C) were key hydrological regimes to induce the spawning success of Chinese sturgeon. However, these conditions did not occur in 2013, 2015 and 2017, thus leading to spawning failure. In 2018 and 2019, although the hydrological regime was suitable, the continuous spawning failure was probably due to too few parent fish. If there is no immediate and substantive recovery measures to increase breeding adults, no recruitment will be provided and this species will follow the extinction of the *Psephurus gladius* (Chinese Paddlefish, [67]). Although more than six million individuals have been continually released into the Yangtze River since 1984, these restocked fish are mostly juveniles or sub-adults, which probably contributed little to the total reproductive stock due to high mortality rate prior to breeding and absence of suitable spawning conditions. Therefore, future conservation measures should focus on both habitat restoration such as optimizing ecological operations of reservoirs to ensure suitable hydrological conditions and matured fish restocking to provide more parent fish in a short time for successful spawning. Fortunately, ecological operation has been proven effective in the conservation of *Acipenser fulvescens*, as water discharge resembling the natural regime resulted in better reproductive performances below the dam with more females present and less time spent on the spawning site [68].

## Conclusions

Our study indicates that eDNA survey based on ddPCR is a highly effective tool to elucidate population processes beyond the presence and absence with high level of sensitivity, even for detecting small populations of large migratory species. For Chinese sturgeon, we demonstrated a strong temporal trend associated with the timing of breeding season. Besides, the eDNA survey reflected that Chinese sturgeon survive in the Yangtze River with an extremely small number of reproductive stock. Therefore, we suggest that the eDNA method could be a valuable and complementary tool to rapidly determine distribution and quantification of endangered aquatic species. Furthermore, eDNA methods will certainly be more applicable through optimizing sampling methods, such as developing a panel of sampling strategies according to season, habitat type and enhancing sampling frequency.

## Supplementary Information

**Additional file 1. Appendix S1**. The specific primers for* Acipenser sinensis* with comparison to the DNA sequences of 18 Acipenseriformes species.** Appendix S2**. The UPGMA phylogenetic tree in Acipenseriformes based on partial D-loop sequence.** Appendix S3**. The eDNA concentration (copies/ul) of three replicates at each site in every month.

## Data Availability

The data that support the findings of this study are available in this published article and its supplementary information files.

## Abbreviations

eDNA: Environmental DNA
ddPCR: Droplet digital PCR
qPCR: Quantitative PCR
UPGMA: Unweighted pair-group method with arithmetic mean
