# Supplementary material for: Using environmental DNA methods to improve detectability in an endangered sturgeon (Acipenser sinensis) monitoring program
Source: BMC Ecol Evol. 2021 Dec 1;21:216. doi: 10.1186/s12862-021-01948-w (PMC8638369; doi:10.1186/s12862-021-01948-w)
Supplement: Supplementary file 1 — Additional file 1. Appendix S1. The specific primers for Acipenser sinensis with comparison to the DNA sequences of 18 Acipenseriformes species. Appendix S2. The UPGMA phylogenetic tree in Acipenseriformes based on partial D-loop sequence. Appendix S3. The eDNA concentration (copies/ul) of three replicates at each site in every month. [file 12862_2021_1948_MOESM1_ESM.docx]

**Appendix S1** The specific primers for *Acipenser sinensis* with comparison to the DNA sequences of 18 Acipenseriformes species.

| **Acipenseriformes species** | **Forward primer** | **Reverse primer** |
| --- | --- | --- |
| *Acipenser sinensis* (AY096295) | GGCAATTTTAATCTGGGTTTCCA | CAAGGACATATATCTAACATCCA |
| *Acipenser* *dabryanus* (AY510085) | GGCACTTTTAATCTGGGTTTCCA | CAGGGACATATATCTAACATCCA |
| *Acipenser baerii* (JQ045341) | GGTATTTTTATTTTAGGTCTCCA | TAATGACATACTCTGGTATACTA |
| *Acipenser fulvescens* (NC030325) | GGTAATCTTTATTTAGGTCTACA | TAATGACATATCCTGAATATCAC |
| *Acipenser gueldenstaedtii* (NC012576) | GGTATTTTTATTTTAGGTCTCCA | TAATGACATATCCTGGATACCAC |
| *Acipenser medirostris* (NC028405) | GGTATTTTTTATTTGGGTTTCCA | CAATGACATATCCTTGACGCTAT |
| *Acipenser mikadoi* (NC031188) | GGTATTTTTTATTTGGGTTTCCA | CAATGACATATCCTTGACGCTAT |
| *Acipenser persicus* (MK213065) | GGTATTTTTATTTTAGGTCTCCA | TAATGACATATTCTGGATACCAC |
| *Acipenser nudiventris* (NC030344) | GGTTATTTTTATTTGGGTCTCCA | TAATGACATACCCTGGATACAGT |
| *Acipenser oxyrinchus* (NC028290) | GGTTATTTTTATTGGGGTC-CCA | CAATGACATATTCTGATATATCA |
| *Acipenser ruthenus* (NC022453) | GGTTATTTTTATTTGGGTTTCCA | CAATGACATATCCAAGATACAAT |
| *Acipenser schrenckii* (NC021757) | GGTATTTTTTATTTGGGTTTCCA | CAATGACATACCCCTGATGTCAC |
| *Acipenser sturio* (NC027417) | GGTTACTTTTATTTGGGTTTCCA | TAATGACATATTCTGATATATTA |
| *Acipenser transmontanus* (NC004743) | GGTATTTTTTATTCGGGTTTCCA | CAATGACATATCCCTGATGTCAC |
| *Huso huso* (NC005252) | GGTTACTCTTATTTCGGTTTCCA | TAATGACATATCCTGGACGCCAT |
| *Psephurus gladius* (AY571339) | TTTAATTATTTTTTCGGTTTCCA | TAATGACATATTCACTGCACTAT |
| *Scaphirhynchus albus* (NC030324) | GGTTATTTTTATTTCGGTTTCCA | CAATGACATACTTTAGACACCAC |
| *Scaphirhynchus platorynchus* (NC030326) | GGTTATTTTTATTTCGGTTTCCA | CAATGACATACTTTAGACACCAC |
| *Scaphirhynchus suttkusi* (NC036060) | GGTTATTTTTATTTCGGTTTCCA | CAATGACATACTTTAGACACCAC |

*Note*: The mismatched nucleotides are highlighted in yellow.

*Acipenser sinensis-*Yiling

*Acipenser sinensis*-Gezhouba

*Acipenser sinensis-*01

*Acipenser sinensis-*02

*Acipenser sinensis-*YanzhiBa

*Acipenser sinensis*-Gulaobei Bay

*Acipenser medirostris* (NC028405)

*Acipenser mikadoi* (NC031188)

*Acipenser schrenckii* (NC021757)

*Acipenser transmontanus* (NC004743)

*Acipenser ruthenus* (NC022453)

*Huso huso* (NC005252)

*Acipenser nudiventris* (NC030344)

*Acipenser fulvescens* (NC030325)

*Acipenser gueldenstaedtii* (NC012576)

*Acipenser baerii* (JQ045341)

*Acipenser persicus* (MK213065)

*Acipenser oxyrinchus* (NC028290)

*Acipenser sturio* (NC027417)

*Scaphirhynchus albus* (NC030324)

*Scaphirhynchus suttkusi* (NC036060)

*Scaphirhynchus platorynchus* (NC030326)

*Psephurus gladius* (AY571339)

0.000

0.050

0.100

0.150

0.200

0.250

*Acipenser dabryanus-*01

*Acipenser dabryanus-*02

**Appendix S2** The UPGMA phylogenetic tree in Acipenseriformes based on partial D-loop sequence.

**Appendix S3** The eDNA concentration (copies/ul) of three replicates at each site in every month.

|  | Gezhou Dam | | | Yiling  Yangtze Bridge | | | YanzhiBa Island | | | Gulaobei Bay | | |
| --- | --- | --- | --- | --- | --- | --- | --- | --- | --- | --- | --- | --- |
| Time | First  sample | Second sample | Third sample | First sample | Second sample | Third sample | First sample | Second sample | Third sample | First sample | Second sample | Third sample |
| 201812 | 0.61 | 0.58 | 1.00 | 2.60 | 1.69 | 1.72 | 0.68 | 0.30 | 0.41 | 0.62 | 0.41 | 0.42 |
| 201906 | 0.00 | 0.00 | 0.00 | 0.00 | 0.00 | 0.00 | 0.00 | 0.00 | 0.00 | 0.00 | 0.00 | 0.00 |
| 201908 | 0.27 | 0.20 | 0.20 | 0.00 | 0.00 | 0.00 | 0.22 | 0.20 | 0.24 | 0.00 | 0.00 | 0.00 |
| 201909 | 0.61 | 0.78 | 0.48 | 0.50 | 0.50 | 0.49 | 1.20 | 0.94 | 1.17 | 0.28 | 0.26 | 0.34 |
| 201910 | 0.14 | 0.16 | 0.12 | 0.00 | 0.00 | 0.00 | 0.29 | 0.43 | 0.28 | 1.39 | 1.40 | 1.40 |
| 201911 | 0.17 | 0.17 | 0.18 | 0.14 | 0.15 | 0.15 | 0.38 | 0.34 | 0.31 | 0.08 | 0.06 | 0.09 |
| 201912 | 0.18 | 0.18 | 0.17 | 0.00 | 0.00 | 0.00 | 0.16 | 0.15 | 0.16 | 0.00 | 0.00 | 0.00 |
| 202001 | 0.08 | 0.07 | 0.09 | 0.00 | 0.00 | 0.00 | 0.00 | 0.00 | 0.00 | 0.00 | 0.00 | 0.00 |
